# Bayesian Nonparametric Modeling for Predicting Dynamic Dependencies in Multiple Object Tracking

**DOI:** 10.3390/s22010388

**Published:** 2022-01-05

**Authors:** Bahman Moraffah, Antonia Papandreou-Suppappola

**Affiliations:** School of Electrical, Computer, and Energy Engineering, Arizona State University, Tempe, AZ 85281, USA

**Keywords:** multiple object tracking, Monte Carlo sampling method, Bayesian nonparametric modeling, dependent Dirichlet process, dependent Pitman–Yor process

## Abstract

The paper considers the problem of tracking an unknown and time-varying number of unlabeled moving objects using multiple unordered measurements with unknown association to the objects. The proposed tracking approach integrates Bayesian nonparametric modeling with Markov chain Monte Carlo methods to estimate the parameters of each object when present in the tracking scene. In particular, we adopt the dependent Dirichlet process (DDP) to learn the multiple object state prior by exploiting inherent dynamic dependencies in the state transition using the dynamic clustering property of the DDP. Using the DDP to draw the mixing measures, Dirichlet process mixtures are used to learn and assign each measurement to its associated object identity. The Bayesian posterior to estimate the target trajectories is efficiently implemented using a Gibbs sampler inference scheme. A second tracking approach is proposed that replaces the DDP with the dependent Pitman–Yor process in order to allow for a higher flexibility in clustering. The improved tracking performance of the new approaches is demonstrated by comparison to the generalized labeled multi-Bernoulli filter.

## 1. Introduction

With emerging technological advances, there is an increasing interest in continuously monitoring and tracking multiple objects in a scene using data from multimodal systems. One of the main challenges in such problems is how to adapt the processing algorithms to rapid changes in the scene. Such changes include different objects entering or leaving the scene, time-variability in environmental or operational conditions and measurements from different sensing modalities with unknown association to the objects. Different methods were proposed in the literature for multiple object tracking such as joint probabilistic data association and multiple hypothesis density filtering [1,2,3]. Most of these methods, however, assume independent state transitions and require known measurement-to-object associations. Other methods involve random finite set (RFS) theory that provide a common mathematical framework for multiple object distributions and has been integrated with probability hypothesis density and multi-Bernoulli filtering [4,5,6,7]. An RFS involves a random number of random and unordered elements and with a cardinality distribution used to estimate the number of elements. Note, however, that RFS based methods for tracking multiple objects require post-processing to pair objects with their estimated state parameters. This is avoided by the generalized labeled multi-Bernoulli (GLMB) filter that uses labeled RFS to estimate the objects identity by assigning distinct labels to different states [5,6,8]. Although the GLMB approach has been successfully used, its implementation requires truncation algorithms as the number of association maps and object labels in computing the filtering density increase exponentially with time. Recently, an efficient implementation was shown to reduce the number of GLMB truncations using Gibbs sampling [7]. However, as approximations are still required, it is difficult to extend the GLMB to practical tracking scenarios with a large number of objects and multimodal sensing systems [9].

In recent years, the ubiquitous influence of Bayesian nonparametric modeling has been well-established as a way to avoid the restrictions of parametric models [10,11]. Infinite-dimensional random measures, such as the Dirichlet process (DP) and Pitman–Yor process (PYP), allow the number of model parameters to vary with the data. As such, they have replaced finite mixture models for clustering, estimation, and inference [12,13,14]. In tracking, the DP was used to learn the number of objects [15] and the hierarchical DP was used to learn changes in the object motion model [16]. However, DP is not adequate for use under time-varying (TV) conditions. In [17,18], TV DP mixtures were used based on a generalized Polya Urn scheme and stationary DP mixture models. A better matched Bayesian nonparametric model is the dependent DP that describes dependency among collections of stochastic processes [19,20,21]. In particular, the dependent DP and mixture model allow for a TV number of clusters for processing batch sequential data [22,23]. As a result, they are well-matched to tracking objects with unknown labels that enter and leave a scene at different times.

In this paper, we propose to incorporate a family of prior distributions to learn unknown time-dependent information in the aforementioned tracking problem. The new multiple object tracking method captures the inherent dynamic dependencies in the state transition. The time-dependent states are the unknown parameters of the multiple objects that are estimated while ensuring that they are assigned to the right object. The object state priors are constructed using the dependent DP and dependent PYP which are shown to have well-defined marginal distributions. The proposed priors along with the likelihood thus provide an efficient way to perform robust inference when integrated with Markov chain Monte Carlo (MCMC) methods. DP mixture models are used to learn and assign each measurement to its associated object identity. The method accurately estimates the dynamically-varying cardinality, identity and state parameters of the multiple objects, with guaranteed convergence.

The rest of the paper is organized as follows. In Section 2, we discuss the multiple object scenario used in the tracking formulation. In Section 3, we first review the dependent DP and then use it to describe our proposed tracking method. The extension to the dependent PYP is provided in Section 4. In Section 5, we provide simulations and performance comparisons with GLMB filtering.

## 2. Multiple Object Tracking Formulation

We consider the problem of tracking multiple objects moving in a field-of-view (FOV) over a time period. The number of objects is unknown and time-varying as objects can enter, exit or stay in the FOV. At any given time step, we want to estimate the number of objects present in the FOV, to associate each measurement to the object it originated from, to estimate the location of each object that is present and to associate each estimated location to its designated object. Note that the solution to such a problem is applicable in many different scenarios, including tracking cars speeding in busy intersections, monitoring air traffic in an airport, and tracking neural activity by estimating the orientation and position of multiple neurons.

If we assume that the *ℓ*th object, *ℓ* = 1,…,N, transitions from time step (k−1) to time step *k* and that the *m*th measurement, *m* = 1,…,M, originates from the *ℓ*th object, then the state space formulation is given by (throughout the paper, row vectors are denoted by boldface lower case letters):

(1) $$\begin{array}{c} x_{\ell ,k}=f\left(x_{\ell ,k-1}\right)+v_{\ell ,k-1} \end{array}$$

(2) $$\begin{array}{c} z_{m,k}=h\left(x_{\ell ,k}\right)+w_{m,k}\;. \end{array}$$

Here, $x_{\ell ,k}$ is the vector of unknown state parameters of the *ℓ*th object, f(·) is the transition function, $v_{\ell ,k}$ is a random vector representing modeling error, $z_{m,k}$ is the *m*th measurement vector with corresponding noise random vector $w_{m,k}$, and h(·) is the measurement function. Using (1) and (2), the state of the *ℓ*th object can be obtained by estimating the posterior probability density function (PDF) $p(x_{\ell ,k}\;|\;z_{m,k})$. This PDF is obtained by first predicting the state using the state transition PDF $p(x_{\ell ,k}\;|\;x_{\ell ,k-1})$ and then updating it using the likelihood $p(z_{m,k}\;|\;x_{\ell ,k})$. However, the complex multiple object tracking problem needs to learn the assumed knowledge before using (1) and (2). Specifically, we consider an unknown TV number $N_{k}$ of objects that enter, leave or stay in the scene at any time. As the identify (or label) of an object is not known a priori, information must be learned to ensure that the previous and current state parameters correspond to the same object before using Equation (1), We also consider a TV number $M_{k}$ of unordered measurements whose associations to the different objects are not known. This information must also be learned before using (2).

The multiple unknown TV information in the resulting problem formulation lead to some inherent dynamic dependencies in the state transition. In particular, if an object transitions between time steps, its label at time step *k* depends on the labels and number of objects at the previous time step (k−1); it also depends on the labels already used by the previously considered objects at the current time step *k*. Thus, the proposed tracking approach must account for these dependencies. To that effect, we exploit Bayesian nonparametric modeling using the dependent DP and dependent PYP to learn the unknown and TV information. The modeled prior PDFs are then integrated with MCMC methods to infer the unknown object state parameters.

**Figure 1:**
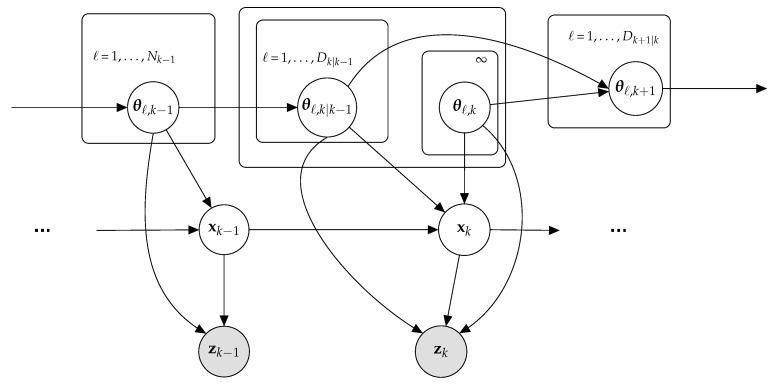
Graphical model capturing dependence in obtaining posterior distribution.

Our proposed formalism is depicted as a cyclic directed graph in Figure 1. One can exploit this graphical model to obtain the posterior distribution of the unknown parameters $x_{k}$ given measurements $z_{k}$ while learning information using parameters $\theta_{k}$, as presented next.

## 3. Multiple Object Tracking with Dependent Dirichlet Process

### 3.1. Dependent Dirichlet Process as Prior

The DP is a Bayesian nonparametric model for random probability measures on an infinite dimensional space [10,14]. The DP *G*∼$\mathrm{DP}(\alpha ,G_{0})$ defines a prior on the space of probability distributions, where α> 0 is the concentration parameter and $G_{0}$ is the base distribution. The strict breaking construction for the DP is given by [24]:

(3) $$\begin{array}{c} G\left(\theta \right)=\sum_{\ell =1}^{\infty }\pi_{\ell }\;\delta \left(\theta -\theta_{\ell }\right), \end{array}$$

where $\theta_{\ell }\;\;\in \;\;\Theta$ are independent and identically distributed random vectors drawn from $G_{0}$, $\pi_{\ell }\;\;\;∼\;\;\;V_{\ell }\;\prod_{l=1}^{\ell -1}\left(1-V_{l}\right)$, and $V_{\ell }\;\;∼\;\;\mathrm{Beta}\left(1,\alpha \right)$ are beta distributed.

The DP can be used to estimate the unknown density of data $x_{\ell }$, *ℓ* = 1,…,N, as $p(x_{\ell })$ = $\int p\left(x_{\ell }\;|\;\theta_{\ell }\right)\;\;dG\left(\theta_{\ell }\right)$, where $p(x_{\ell }\;\;|\;\;\theta_{\ell })$ is the data distribution indexed by $\theta_{\ell }$ and the DP *G*∼$\mathrm{DP}(\alpha ,G_{0})$ is its underlying probability random measure. The DP is also useful for clustering data using mixture models without prior knowledge on the number of clusters. Specifically, data samples $x_{\ell }$ form a cluster if their modeled distribution $p(x_{\ell }\;|\;\theta_{\ell })$ is parameterized by the same parameter $\theta_{\ell }$ drawn from *G*∼$\mathrm{DP}(\alpha ,G_{0})$. This follows from the fact that DP is discrete (with probability one) and the same value of $\theta_{\ell }$ can be drawn multiple times. The DP mixture (DPM) model is a mixture model with a countably infinite number of clusters. The clustering is learned by probabilistically assigning data to clusters proportional to the number of elements in that cluster. Given DP parameter set $\Theta_{\ell -1}$ = $\left\{\theta_{1},\ldots ,\theta_{\ell -1}\right\}$, the predictive distribution of the next $\theta_{\ell }$ drawn from the DP is given by [14]:

(4) $$\begin{array}{c} p\left(\theta_{\ell }\;|\;\Theta_{\ell -1},G_{0},\alpha \right)=P^{\left(1\right)}\;G_{0}\left(\theta_{\ell }\right)+P^{\left(2\right)}\;\sum_{j=1}^{\ell -1}\delta \left(\theta_{\ell }-\theta_{j}\right) \end{array}$$

with probabilities $P^{\left(1\right)}=\frac{\alpha }{\ell -1+\alpha }\;\;\mathrm{and}\;\;P^{\left(2\right)}=\frac{1}{\ell -1+\alpha }$.

Since $\Theta_{\ell -1}$ is infinitely exchangeable, the probability of generating the set in any order is the same [25]. Then, the *j*th cluster is obtained as the set of $\ell_{j}$ draws from DP that result in the same unique parameter $\theta_{j}^{🟉}$. Thus, Equation (4) can also be written as:

$$\begin{array}{c} p\left(\theta_{\ell }\;|\;\Theta_{\ell -1},G_{0},\alpha \right)=P^{\left(1\right)}\;G_{0}\left(\theta_{\ell }\right)+P^{\left(2\right)}\;\sum_{j=1}^{\ell -1}\ell_{j}\;\delta \left(\theta_{\ell }-\theta_{j}^{🟉}\right)\;. \end{array}$$

When clustering is required under TV conditions, the DP assumption of exchangeability no longer holds. In such scenarios, the DDP provides a well-matched model as it allows for dynamic clustering [20]. The DDP mixture model and clustering property may be obtained similarly to the DP. The main difference is that the DDP cluster parameter set $\Theta_{k}$ varies with time, allowing for clusters to transition between time steps or for new clusters to form at any time [21]. We thus make use of the DDP and its properties to solve the TV multiple object tracking problem.

### 3.2. Construction of DDP Prior for State Prediction

The proposed DDP-based State Transitioning Prior (DDP-STP) approach exploits the dynamic clustering property of the DDP prior to model the dynamic dependencies in the state transition formulation. These dynamic dependencies arise inherently as: (a) the number of objects present at time step *k* depends on the number of objects present at the previous time step (k−1); and (b) the clustering index of the *ℓ*th object state at time step *k* depends on the clustering indices of the previously clustered (ℓ−1) object states at the same time step *k*. We use the DDP prior to learn the dynamic clustering of object states to ensure that correctly identified object states are used in Equation (1) if the object remains in the scene. The DDP learned cluster parameter $\theta_{\ell ,k-1}$ is assumed to be assigned to the *ℓ*th object with state parameter vector $x_{\ell ,k-1}$ at time step (k−1). This prior is designed such that (based on the DDP definition) we have a DP at each time step. Thus, a DP is used to model a new object entering the scene without requiring any prior knowledge on the expected number of objects. The DDP-based state prior construction algorithm is described next in detail and summarized in Algorithm 1 [26].

As the algorithm is recursive, we provide (i) the parameters that are assumed available at time step (k−1), (ii) the transitioning of the parameters from time step (k−1) to time step *k*, and (iii) the development of the object state transition model to form the multiple object state prior at time step *k*. Note that, as a nonparametric algorithm, the number of parameters varies with time as new measurements become available [11]. Note, also, that the recursive algorithm is initialized by drawing $\theta_{\ell ,0}$ from $\mathrm{DP}(\alpha ,G_{0})$.

(i) *Parameters available at the previous time step*:
  At time step (k−1), we assume that $N_{k-1}$ objects are present in the tracking scene and that there are $D_{k-1}\;\;\;\leq \;\;\;N_{k-1}$ non-empty (unique) DDP clusters. As unique clusters can include more than one object, multiple objects can be related to the same cluster parameter. We also assume that the following parameters are available at time step (k−1).
  – Set of object state vectors, $X_{N_{k-1},k-1}$ = $\left\{x_{1,k-1},\ldots ,x_{N_{k-1},k-1}\right\}$
  – Set of DDP cluster parameters for object states, $\Theta_{N_{k-1},k-1}$ = $\left\{\theta_{1,k-1},\ldots ,\theta_{N_{k-1},k-1}\right\}$
  – Set of unique DDP cluster parameters, $\Theta_{D_{k-1},k-1}^{🟉}\;\;\subseteq \;\Theta_{N_{k-1},k-1}$
  – Cardinality of *l*th unique cluster, $q_{l,k-1}^{🟉}$ = ${\left[q_{k-1}^{🟉}\right]}_{l}$, *l* = $1,\ldots ,D_{k-1}$
  – Cluster label indicator, $c_{l,k}$, *l* = $1,\ldots ,D_{k-1}$ and set $C_{D_{k-1},k-1}$ = $\left\{c_{1,k-1},\ldots ,c_{D_{k-1},k-1}\right\}$
(ii) *Transitioning between time steps.*
  From time step (k−1) to time step *k*, objects may leave the scene or remain (survive) in the scene. We model this transition using an object survival indicator $s_{\ell ,k|k-1}$ that is drawn from a Bernoulli process whose parameter is the probability of object survival $P_{\ell ,k|k-1}$. If $s_{\ell ,k|k-1}$ = 1, the *ℓ*th object with state $x_{\ell ,k-1}$ remains in the scene with probability $P_{\ell ,k|k-1}$; if $s_{\ell ,k|k-1}$ = 0, the object leaves the scene with probability $\left(1-P_{\ell ,k|k-1}\right)$. The total number of objects that transitioned is given by $N_{k|k-1}$ = $\sum_{\ell =1}^{N_{k-1}}s_{\ell ,k|k-1}$.

**Algorithm 1** Construction of the prior distribution of DDP-STP(i) *Available parameters at time step (k−1)* – Object state parameter $x_{\ell ,k-1}$, *ℓ* = $1,\ldots ,N_{k-1}$, set $X_{N_{k-1},k-1}$ – Cluster parameter $\theta_{\ell ,k-1}$, *ℓ* = $1,\ldots ,N_{k-1}$, for *ℓ*th object, set $\Theta_{N_{k-1},k-1}$ – Parameter of unique cluster $\theta_{l,k-1}^{🟉}$, *l* = $1,\ldots ,D_{k-1}$, set $\Theta_{D_{k-1},k-1}^{🟉}$ – Cluster label indicator $c_{l,k-1}$, *l* = $1,\ldots ,D_{k-1}$ and set $C_{D_{k-1},k-1}$ – Cardinality of *l*th unique cluster $q_{l,k-1}^{🟉}$, *l* = $1,\ldots ,D_{k-1}$(ii) *Transitioning from time step (k−1) to k* – Draw object survival indicator $s_{\ell ,k|k-1}$∼$\mathrm{Bernoulli}(P_{\ell ,k|k-1})$, *ℓ* = $1,\ldots ,N_{k-1}$ – If $s_{\ell ,k|k-1}$ = 1, the *ℓ*th object survives; if $s_{\ell ,k|k-1}$ = 0, it leaves the scene – Compute number of transitioned objects $N_{k|k-1}$ = $\sum_{\ell =1}^{N_{k-1}}s_{\ell ,k|k-1}$ – Denote cardinality of *l*th cluster, *l* = $1,\ldots ,D_{k-1}$, after transitioning by $q_{l,k|k-1}$ – If $q_{l,k|k-1}$≥ 1, cluster survival indicator $\lambda_{l,k|k-1}$ = 1; if $q_{l,k|k-1}$ = 0, $\lambda_{l,k|k-1}$ = 0 – Compute number of unique clusters to $D_{k|k-1}$ = $\sum_{l=1}^{D_{k-1}}\lambda_{l,k|k-1}$ – Denote cardinality of *l*th transitioned cluster by $q_{l,k|k-1}^{🟉}$, *l* = $1,\ldots ,D_{k|k-1}$ – Denote parameter of transitioned cluster by $\theta_{l,k|k-1}^{🟉}$, *l* = $1,\ldots ,D_{k|k-1}$(iii) *Current time step k*  **for** *ℓ* = 1 **to** $D_{k|k-1}$ **do**   **if** Case 1 (on page 6) **then**    Draw $x_{\ell ,k}$ from the prior PDF in (6) with probability $P_{k}^{\left(1\right)}$ in (5)   **else if** Case 2 (on page 6) **then**    Draw $\theta_{\ell ,k}$ from $p(\theta_{\ell ,k}\;|\;\theta_{\ell ,k-1}^{🟉})$    Draw $x_{\ell ,k}$ from the prior PDF in (8) with probability $P_{k}^{\left(2\right)}$ in (7)   **else if** Case 3 (on page 7) **then**    Draw $\theta_{\ell ,k}∼G_{0}$ following $\mathrm{DP}(\alpha ,G_{0})$    Draw $x_{\ell ,k}$ from the PDF in (10) with probability $P_{k}^{\left(3\right)}$ in (9)   **end if**  **end for**  Update number of objects $N_{k}$ using $N_{k|k-1}$ and number of new objects under Case 3  Update *l*th unique cluster cardinality $q_{l,k}^{🟉}$ and parameter $\theta_{l,k}^{🟉}$  **return** $X_{N_{k},k}$, $\Theta_{N_{k},k}$

If all objects in a cluster leave the scene, we assume that the cluster no longer exists. If at least one object from the cluster remains in the scene, then the cluster survives and transitions to time step *k*. After transitioning, we denote the new cardinality of the $D_{k-1}$ clusters by $q_{l,k|k-1}$, *l* = $1,\ldots ,D_{k-1}$; if the *l*th cluster is empty, we set $q_{l,k|k-1}$ = 0. In order to keep track of the transitioned clusters, we define a cluster survival indicator $\lambda_{l,k|k-1}$. We set $\lambda_{l,k|k-1}$ = 1 if $q_{l,k|k-1}\;\;\geq \;\;1$ and $\lambda_{l,k|k-1}$ = 0 if $q_{l,k|k-1}$ = 0. Using this indicator, the number of transitioned clusters is $D_{k|k-1}$ = $\sum_{l=1}^{D_{k-1}}\lambda_{l,k|k-1}$. We denote by $q_{l,k|k-1}^{🟉}$ and $\theta_{l,k|k-1}^{🟉}$ the cardinality and parameter, respectively, of the *l*th unique transitioned cluster, *l* = $1,\ldots ,D_{k|k-1}$.

(iii) *State prediction at current time step.*
  We identify the cluster parameter $\theta_{\ell ,k}$ for the *ℓ*th object present at time step *k* following three case scenarios. In Case 1, the *ℓ*th object survived, *ℓ* = $1,\ldots ,N_{k|k-1}$, from a transitioned cluster that is already occupied by at least one of the first (ℓ−1) transitioned objects. In Case 2, the *ℓ*th object survived, *ℓ* = $1,\ldots ,N_{k|k-1}$, from a cluster not yet transitioned. In Case 3, a new object enters the scene and a new cluster is generated. The prior state PDF obtained in each case is discussed next.

Case 1: The *ℓ*th object transitioned in a cluster already occupied by at least one of the (ℓ−1) previously clustered objects. As the cluster label indicator set $C_{D_{k},k}$ induces an infinite exchangeable random partition, the *ℓ*th object is assumed the last to be clustered. The object selects an existing transitioned cluster with probability $P_{k}^{\left(1\right)}$ = $\mathrm{Pr}\left(\mathrm{select}\;l\mathrm{th}\;\mathrm{cluster},\;l\leq D_{k|k-1}|\Theta_{\ell -1,k}\right)$, where:

(5) $$\begin{array}{c} P_{k}^{\left(1\right)}=\frac{q_{l,k}+\sum_{j=1}^{D_{k|k-1}}q_{j,k|k-1}^{🟉}\;\lambda_{j,k|k-1}\;\delta \left(c_{l,k}-c_{j,k}\right)}{\left(\ell -1\right)+\alpha +\sum_{i=1}^{\ell -1}\sum_{j=1}^{D_{k|k-1}}q_{j,k|k-1}^{🟉}\;\lambda_{j,k|k-1}\;\delta \left(c_{i,k}-c_{j,k}\right)}\;. \end{array}$$

Thus, the probability depends both on the number of objects in the *l*th cluster at time *k* and on the number of objects that survived in the same cluster from time (k−1). With probability $P_{k}^{\left(1\right)}$, the state prior PDF of the *ℓ*th object is given by:

(6) $$\begin{array}{c} p_{1}\left(x_{\ell ,k}|X_{\ell -1,k},X_{\ell ,k-1},\Theta_{D_{k|k-1},k|k-1}^{🟉},\Theta_{\ell -1,k}\right)\propto p\left(x_{\ell ,k}\;|\;x_{\ell ,k-1},\theta_{\ell ,k}\right), \end{array}$$

where: $p(x_{\ell ,k}\;|\;x_{\ell ,k-1},\theta_{\ell ,k})$ is obtained from (1), and is selected from an infinite number of Gaussian PDFs with parameter $\theta_{\ell ,k}$. It is worth mentioning that we only choose Gaussian PDFs for the sake of simplicity and one can choose any valid distribution without compromising the theory.

Case 2: The *ℓ*th transitioned object is in a cluster that has not yet been selected by the previous (ℓ−1) objects. The object transitions in this cluster with the probability: $P_{k}^{\left(2\right)}$ = $\mathrm{Pr}\left(\mathrm{select}\;l\mathrm{th}\;\mathrm{cluster},l\;\leq \;D_{k|k-1}|\Theta_{\ell -1,k}\right)$, where:

(7) $$\begin{array}{c} P_{k}^{\left(2\right)}=\frac{\sum_{j=1}^{D_{k|k-1}}q_{j,k|k-1}^{🟉}\;\lambda_{j,k|k-1}\;\delta \left(c_{l,k}-c_{j,k}\right)}{\left(\ell -1\right)+\alpha +\sum_{i=1}^{\ell -1}\sum_{j=1}^{D_{k|k-1}}q_{j,k|k-1}^{🟉}\;\lambda_{j,k|k-1}\;\delta \left(c_{i,k}-c_{j,k}\right)}\;. \end{array}$$

The cluster parameter $\theta_{\ell ,k}$ associated with the *ℓ*th object is drawn from the DDP prior PDF $p(\theta_{\ell ,k}\;|\;\theta_{\ell ,k-1}^{🟉})$ that evolves through transition equation $\theta_{\ell ,k}$ = $\theta_{\ell ,k-1}^{🟉}+\nu_{k-1}$ where $\nu_{k-1}$ is a known time-dependent Gaussian variable. With probability $P_{k}^{\left(2\right)}$, the state prior PDF of the *ℓ*th object is given by:

(8) $$\begin{array}{c} \;p_{2}\left(x_{\ell ,k}|X_{\ell -1,k},X_{\ell ,k-1},\Theta_{D_{k|k-1},k|k-1}^{🟉},\Theta_{\ell -1,k}\right)\propto p\left(x_{\ell ,k}\;|\;x_{\ell ,k-1},\theta_{\ell ,k}\right)\;p\left(\theta_{\ell ,k}\;|\;\theta_{\ell ,k-1}^{🟉}\right)\;. \end{array}$$

Case 3: As the *ℓ*th object enters the scene at time *k*, it does not belong to an existing cluster. A new cluster is formed with parameter $\theta_{\ell ,k}\;∼\;G_{0}$ obtained from the base distribution of $\mathrm{DP}(\alpha ,G_{0})$. The object selects this cluster with probability

(9) $$\begin{array}{ccc} & & \;P_{k}^{\left(3\right)}=\mathrm{Pr}\left(\mathrm{new}\;\mathrm{cluster}|\Theta_{\ell -1,k}\right)=\frac{\alpha }{\left(\ell -1\right)+\alpha +\sum_{i=1}^{\ell -1}\sum_{j=1}^{D_{k|k-1}}q_{j,k|k-1}^{🟉}\;\lambda_{j,k|k-1}\;\delta \left(c_{i,k}-c_{j,k}\right)}\;. \end{array}$$

With probability $P_{k}^{\left(3\right)}$, the state PDF is obtained as:

(10) $$\begin{array}{c} p_{3}\left(x_{\ell ,k}\right)=\int_{\theta }p\left(x_{\ell ,k}|\theta \right)\;dG_{0}\left(\theta \right)\;. \end{array}$$

Thus, the predicted object state parameter distribution at time step *k* is given by:

(11) $$\begin{array}{c} p\left(x_{\ell ,k}\;|\;x_{\ell ,k-1},\theta_{\ell ,k},\theta_{\ell ,k-1}^{🟉}\right)\propto \left\{\begin{array}{cc} p(x_{\ell ,k}\;|\;x_{\ell ,k-1},\theta_{\ell ,k})\;, & \mathrm{if}\;\mathrm{Case}\;1\\ p\left(x_{\ell ,k}\;|\;x_{\ell ,k-1},\theta_{\ell ,k}\right)\;p\left(\theta_{\ell ,k}\;|\;\theta_{\ell ,k-1}^{🟉}\right), & \mathrm{if}\;\mathrm{Case}\;2\\ p_{3}\left(x_{\ell ,k}\right), & \mathrm{if}\;\mathrm{Case}\;3 \end{array}\right. \end{array}$$

For Cases 1 and 2, the object cardinality at time step *k* is set to $N_{k}$ = $N_{k|k-1}$. Furthermore, the cluster parameter $\theta_{\ell ,k}$ is set to $\theta_{l,k|k-1}^{🟉}$, if the *l*th transitioned cluster includes the *ℓ*th transitioned object. For Case 3, $N_{k}$ is given by $N_{k|k-1}$ plus the number of new objects entering scene. Before the next time step, we denote the *l*th unique cluster cardinality and parameter by $q_{l,k}^{🟉}$, $c_{l,k}$ and $\theta_{l,k}^{🟉}$, respectively.

The DDP in Cases 1–3 defines marginal DPs at each time step *k*, given the DDP configurations at time step (k−1). We denote this as: ${\mathrm{DDP}\text{-}\mathrm{STP}}_{k}\;|\;{\mathrm{DDP}\text{-}\mathrm{STP}}_{k-1}$∼$\mathrm{DDP}\left(\alpha ,H\right)$, with the base distribution given by

(12) $$\begin{array}{c} \;H\left(\theta_{\ell ,k}\right)=P_{k}^{\left(1\right)}\;\sum_{\begin{array}{c} j=1\\ \theta_{j,k}\in \Theta_{D_{k},k} \end{array}}^{D_{k}}\;\delta \left(\theta_{\ell ,k}-\theta_{j,k}\right)+P_{k}^{\left(2\right)}\;\sum_{\begin{array}{c} j=1\\ \theta_{j,k}\in \Theta_{D_{k},k|k-1}^{🟉}\setminus \Theta_{D_{k},k} \end{array}}^{D_{k}}\;p\left(\theta_{\ell ,k}\;|\;\theta_{\ell ,k-1}^{🟉}\right)\;\delta \left(\theta_{\ell ,k}-\theta_{j,k}\right)+P_{k}^{\left(3\right)}\;G_{0}\left(\theta_{\ell ,k}\right) \end{array}$$

Note that the DDP-based model also allows for the variation and labeling of clusters as it is defined in the space of partitions.

### 3.3. Learning Measurement Model for State Update

The predicted state parameter distributions at time step *k* in (11) needs to be updated using the available measurements $z_{m,k}$, *m* = $1,\ldots ,M_{k}$. The updated distribution is then used to estimate the time-dependent object cardinality and to infer posterior distributions using MCMC. We assume that each measurement is generated by only one object, and thus belongs to only one cluster, and is independent of other measurements. We can thus exploit Dirichlet process mixtures (DPMs) with the base distribution drawn from the DDP in Algorithm 1 to cluster the measurements. Note that the measurement vectors are unordered in that the *m*th measurement is not necessarily associated to the *ℓ*th object state. As the objects are already labeled from their DDP clustering, the DPM model is used to learn the association between each measurement and its corresponding object label. The likelihood distribution is inferred from:

(13) $$\begin{array}{c} z_{m,k}\;|\;x_{\ell ,k},\psi_{m,k}∼p\left(z_{m,k}\;|\;x_{\ell ,k},\psi_{m,k}\right) \end{array}$$

where $p(z_{m,k}\;|\;x_{\ell ,k},\theta_{\ell ,k},\psi_{m,k})$ depends on DDP(α,H), on the measurement likelihood function $p(z_{m,k}\;|\;x_{\ell ,k},\psi_{m,k})$ in (2), and on $\mathrm{DP}(\beta ,H^{\prime })$ for the measurement parameters ψ. Algorithm 2 summarizes the mixing process that associates measurements to objects. Note that, as a result of using DPMs, clutter can be separated from measurements that originate from objects without requiring prior knowledge of the clutter statistics. This ensures that performance does not deteriorate when tracking in clutter.

**Algorithm 2** Infinite mixture model for measurement-to-object association **Input**: $\left\{z_{1,k},\ldots ,z_{M_{k},k}\right\}$, measurements From construction of prior distribution from Algorithm 1 **Input**: Object state vectors $\left\{x_{1,k},x_{2,k},\ldots \right\}$ **Input**: Cluster parameter vectors $\left\{\theta_{1,k},\theta_{2,k},\ldots \right\}$ **Input**: Cluster label indicators **At time**
*k*: **for**
*m* = 1 $M_{k}$ **do**  Draw $z_{m,k}|x_{\ell ,k},\psi_{m,k}$ from Equation (13)  **return** $C_{D_{k},k}$, induced cluster assignment indicators **end for** **return**
$D_{k}$ (number of clusters) and $CA_{k}$ **return** posterior of $z_{m,k}|x_{\ell ,k},\theta_{\ell ,k}$, *m* = $1,\ldots ,M_{k}$

The Bayesian posterior to estimate the target trajectories is efficiently implemented using a Gibbs sampler inference scheme. The scheme iterates between sampling the object states and the dynamic DDP parameters, and it is based on the discreteness of the DDP [20,27]. Marginalizing out all parameters, the Bayesian posterior is:

(14) $$\begin{array}{c} p\left(x_{\ell ,k}\;|\;Z_{k}\right)=\int p\left(x_{\ell ,k}\;|\;Z_{k},\Theta_{D_{k},k},\Psi_{k}\right)\;\;dG\left(\Theta_{D_{k},k}\;|\;Z_{k}\right)\;dG\left(\Psi_{k}\;|\;Z_{k}\right) \end{array}$$

where $G(\Theta_{D_{k},k}\;\;|\;\;Z_{k})$ is the cluster parameter posterior distribution given the measurements and $G(\Psi_{k}\;\;|\;\;Z_{k})$ is the parameter posterior distribution given the measurements. As the direct computation of Equation (14) is not realizable [12,28], we exploit Gibbs sampling to predict $x_{\ell ,k}$ given the measurements. Note that it can be shown that the posterior predictive distribution of state parameters is given by:

(15) $$\begin{array}{c} \;\pi \left(\theta_{\ell ,k}|\Theta_{D_{k},k}\right)=P_{k}^{\left(1\right)}\;\sum_{\begin{array}{c} j=1,j\neq \ell \\ \theta_{j,k}\in \Theta_{D_{k},k} \end{array}}^{D_{k}}\;\delta \left(\theta_{\ell ,k}-\theta_{j,k}\right)+P_{k}^{\left(2\right)}\;\sum_{\begin{array}{c} j=1,j\neq \ell \\ \theta_{j,k}\in \Theta_{D_{k|k-1},k|k-1}^{🟉}\setminus \Theta_{D_{k},k} \end{array}}^{D_{k|k-1}}\;p\left(\theta_{\ell ,k}^{🟉}\;|\;\theta_{\ell ,k-1}\right)\;\delta \left(\theta_{\ell ,k}-\theta_{j,k}\right)+P_{k}^{\left(3\right)}\;G_{0}\left(\theta_{\ell ,k}\right). \end{array}$$

The posterior distribution of the states given the parameters and measurements, $p(x_{\ell ,k}\;|\;Z_{k},\Theta_{D_{k},k},\Psi_{k})$ is evaluated as:

(16) $$\begin{array}{c} p\left(x_{\ell ,k}\;|\;Z_{k},\Theta_{D_{k},k},\Psi_{k}\right)\propto p\left(z_{m,k}\;|\;x_{\ell ,k},\psi_{m,k}\right)p\left(x_{\ell ,k}\;|\;Z_{k-1},\Theta_{D_{k},k},\Psi_{k-1}\right). \end{array}$$

The Gibbs sampler distribution for state parameters $\Theta_{D_{k},k}$ given the measurements is

(17) $$\begin{array}{c} \theta_{\ell ,k}|\Theta_{k}^{\left(-\ell \right)},Z_{k}\;∼\;\sum_{l=1}^{D_{k}}\xi_{l,k}\;\delta \left(\theta_{\ell ,k}-\theta_{l,k}\right)+\sum_{\begin{array}{c} l=1\\ l\notin C_{D_{k},k} \end{array}}^{D_{k|k-1}}\beta_{l,k}\;p\left(z_{\ell ,k}|x_{l,k},\theta_{l,k}\right)+\gamma_{\ell ,k}\;H_{\ell ,k}\left(\theta_{\ell ,k}\right), \end{array}$$

where $\Theta_{k}^{\left(-\ell \right)}$ = $\left\{\theta_{1,k},\theta_{2,k},\;\ldots ,\;\theta_{\ell -1,k},\theta_{\ell +1,k},\;\ldots ,\;\theta_{D_{k|k-1},k}\right\}$, $\gamma_{\ell ,k}$ = $1-\sum_{l=1}^{D_{k}}\xi_{l,k}-\sum_{\begin{array}{c} l=1\\ l\notin C_{D_{k},k} \end{array}}^{D_{k|k-1}}\beta_{l,k}$,

$$\begin{array}{ccc} & & \;\xi_{l,k}=p\left(z_{\ell ,k}|x_{l,k},\theta_{l,k}\right)\frac{q_{l,k}+\sum_{j=1}^{D_{k|k-1}}q_{j,k|k-1}^{🟉}\;\lambda_{j,k|k-1}\;\delta \left(c_{l,k}-c_{j,k}\right)}{\left(\ell -1\right)+\alpha +\sum_{i=1}^{\ell -1}\sum_{j=1}^{D_{k|k-1}}q_{j,k|k-1}^{🟉}\;\lambda_{j,k|k-1}\;\delta \left(c_{i,k}-c_{j,k}\right)}\\ & & \;\beta_{l,k}=\frac{\sum_{\begin{array}{c} j=1\\ j\notin C_{D_{k},k} \end{array}}^{D_{k|k-1}}q_{j,k|k-1}^{🟉}\;\lambda_{j,k|k-1}}{\left(\ell -1\right)+\alpha +\sum_{i=1}^{\ell -1}\sum_{j=1}^{D_{k|k-1}}q_{j,k|k-1}^{🟉}\;\lambda_{j,k|k-1}\;\delta \left(c_{i,k}-c_{j,k}\right)}\;. \end{array}$$

Furthermore, $H_{\ell ,k}\left(\theta_{\ell ,k}\right)\;\;\propto \;\;p\left(z_{\ell ,k}\;|\;x_{j,k},\theta_{j,k}\right)\;G_{0}\left(\theta_{\ell ,k}\right)$ and $G_{0}$ is the base distribution on $\theta_{\ell ,k}$. The derivation is provided in [28]. It can be shown that the Gibbs sampler for $G(\Psi_{k}\;\;|\;\;Z_{k})$ is:

(18) $$\begin{array}{c} \psi_{m,k}|\;\;Z_{k},\Psi_{k}^{\left(-m\right)}∼\sum_{s\neq m}q_{ms}\delta \left(\theta_{m,k}-\theta_{m,s}\right)+r_{m}H_{m,k} \end{array}$$

where $\Psi_{k}^{\left(-m\right)}$ is the set of all parameters excluding the *m*th measurement. Furthermore, $q_{ms}\propto p\left(z_{m,k}\;|\;x_{\ell ,k},\psi_{m,s}\right)$, $H_{m,k}$ is the distribution of $\psi \;|\;z_{m,k},H^{\prime }$, and $\sum_{s\neq m}q_{ms}+r_{m}=1$.

### 3.4. DDP-STP Approach Properties

*Convergence*: In the Gibbs sampler, it can be shown that the cluster parameter transition kernel converges to the posterior distribution for almost all initial conditions $\theta_{\ell ,0}$. If after *n* iterations of the algorithm, $A_{k}^{\left(n\right)}\left(\Theta_{D_{k},k}\;|\;\theta_{\ell ,0}\right)$ is the transition kernel for the Markov chain starting at $\theta_{\ell ,0}$ and stopping in the set $\Theta_{D_{k},k}$, then it can be shown to converge to the posterior $G(\Theta_{D_{k},k}\;|\;Z_{k})$ given measurements $Z_{k}$ at time step *k*. Specifically, $\left|\right|A_{k}^{\left(n\right)}\left(\Theta_{D_{k},k}\;|\;\theta_{\ell ,0}\right)-G\left(\Theta_{D_{k},k}\;|\;Z_{k}\right){\left|\right|}_{\mathrm{TVN}}\to 0\;\mathrm{as}\;n\to \infty$, for almost all initial conditions in the total variation norm (TVN) (see [29,30] in relation to the Gaussian distribution). The proof of convergence can be found in [28].

*Exchangeability*: The infinite exchangeable random partition induced by $C_{D_{k},k}$ at time *k* follows the exchangeable partition probability function [25]:

$$\begin{array}{c} p_{N_{k}}\left(q_{k}^{🟉}\right)=\frac{\alpha^{D_{k}}}{\alpha^{\left[N_{k}\right]}}\prod_{j=1}^{D_{k}}\left(q_{j,k}^{🟉}-1\right) \end{array}$$

where $q_{k}^{🟉}$ = ${\left[q_{1,k}^{🟉}\;\ldots \;q_{D_{k},k}^{🟉}\right]}^{T}$, $q_{l,k}^{🟉}$ is the cardinality of the cluster with assignment indicator $c_{l,k}\;\in \;C_{D_{k},k}$, and $\alpha^{\left[n\right]}$ = α(α+1)⋯(α+n−1). Due to the variability of $N_{k}$, there is an important relationship between the partitions based on $N_{k}-1$ and $N_{k}$. In particular, given the configuration at time (k−1), $p_{N_{k}-1}\left(q_{k}^{🟉}\right)$ = $\sum_{j=1}^{D_{k}}p_{N_{k}}\left(q_{j,k}^{🟉}\right)+p_{N_{k}}\left([q_{k}^{🟉}\;1]\right)$, where $q_{j,k}^{🟉}$ = $\left[q_{1,k}^{🟉}\;\ldots \;\left(q_{j,k}^{🟉}+1\right)\;q_{j+1,k}^{🟉}\;\ldots \;q_{D_{k},k}^{🟉}\right]$. This relationship entails a notion of consistency of the partitions in the distribution sense, and it holds due to the Markov property of the process given the configuration at time (k−1).

*Consistency*: We consider $r_{\theta_{0}}$ to be the true measurement density with probability measure $R_{\theta_{0}}$. Then, if $r_{\theta_{0}}$ is in the Kullback–Leibler (KL) support of the prior distribution in the space of all parameters [31], then the posterior distribution $G(\cdot \;|\;Z_{k})$ can be shown to be weakly consistent at $r_{\theta_{0}}$. It is also important to investigate the posterior contraction rate as it is highly related to posterior consistency. This rate shows how fast the posterior distribution approaches the true parameters from which the measurements are generated. As detailed in [28], the contraction rate matches the minimax rate for density estimators. Hence, the DDP prior constructed through the proposed model achieves the optimal frequentist rate.

## 4. Tracking with Dependent Pitman–Yor Process

Another Bayesian nonparametric model for random probability measures is the Pitman–Yor process (PYP) *G*∼$\mathrm{PYP}(d,\alpha ,G_{0})$ [13]. In addition to the concentration parameter α and based distribution $G_{0}$ offered by the DP, the PYP includes the discount parameter d∈(0,1), with α>−d. When *d* = 0, the PYP simplifies to $\mathrm{DP}(\alpha ,G_{0})$. The discount parameter allows for a higher flexibility in clustering as the number of unique clusters under a PYP prior grows much more rapidly than a DP prior [32]. The stick breaking construction for the PYP is similar to (3) but with beta distributed parameters $V_{\ell }\;\;∼\;\;\mathrm{Beta}\left(1-d,\alpha +\ell \;d\right)$.

This flexibility in clustering allows us to extend the tracking algorithm in Section 3 by replacing the dependent DP by the dependent PYP (DPYP). The PYP model has a higher probability of having a large number of unique clusters; also, clusters with only a small number of objects have a lower probability of selecting new objects. In particular, for $N_{k}$ objects to be clustered, whereas the expected number of unique clusters used by the DP during transitioning is $\alpha \log(N_{k})$, the number used by the PYP follows the power law $\alpha N_{k}^{d}$. The more flexible DPYP model is better matched to increased TV activity in objects entering or staying in the scene at each time step.

The proposed DPYP state transitioning prior (DPY-STP) tracking approach is developed similarly to the DDP-based approach. The main difference is in the object clustering when constructing the state prior distribution. In particular, the probability of an object selecting a particular cluster following Cases 1–3 in Section 3.2 are now given as follows [33]. Under Case 1, the transitioned object selects an existing transitioned cluster with probability

$$\begin{array}{c} P_{k}^{\left(1\right)}=\frac{q_{l,k}+\sum_{j=1}^{D_{k|k-1}}q_{j,k|k-1}^{🟉}\;\lambda_{j,k|k-1}\;\delta \left(c_{l,k}-c_{j,k}\right)-d}{\sum_{i=1}^{\ell -1}q_{i,k}+\alpha +\sum_{i=1}^{\ell -1}\sum_{j=1}^{D_{k|k-1}}q_{j,k|k-1}^{🟉}\;\lambda_{j,k|k-1}\;\delta \left(c_{i,k}-c_{j,k}\right)}\;. \end{array}$$

Under Case 2, the transitioned object selects a cluster not yet selected with the probability:

$$\begin{array}{c} P_{k}^{\left(2\right)}=\frac{\sum_{j=1}^{D_{k|k-1}}q_{j,k|k-1}^{🟉}\;\lambda_{j,k|k-1}\;\delta \left(c_{l,k}-c_{j,k}\right)-d}{\sum_{i=1}^{\ell -1}q_{i,k}+\alpha +\sum_{i=1}^{\ell -1}\sum_{j=1}^{D_{k|k-1}}q_{j,k|k-1}^{🟉}\;\lambda_{j,k|k-1}\;\delta \left(c_{i,k}-c_{j,k}\right)}\;. \end{array}$$

Under Case 3, a new cluster is generated with the probability:

$$\begin{array}{c} P_{k}^{\left(3\right)}=\frac{\alpha +d\;D_{k}^{\left(\ell -1\right)}}{\sum_{i=1}^{\ell -1}q_{i,k}+\alpha +\sum_{i=1}^{\ell -1}\sum_{j=1}^{D_{k|k-1}}q_{j,k|k-1}^{🟉}\;\lambda_{j,k|k-1}\;\delta \left(c_{i,k}-c_{j,k}\right)}\;. \end{array}$$

where $D_{k}^{\left(\ell -1\right)}$ is the total number of clusters used by the previous (ℓ−1) objects.

The main difference in the learning algorithm to update the object states in Section 3.3 is that the mixing measure is drawn from the DPYP. Both the DDP-STP and DPY-STP algorithms use DPMs to learn the measurement-to-object associations.

## 5. Simulation Results

We use simulations to demonstrate the performance of the proposed DDP-STP and DPY-STP tracking methods. We also compare them with the generalized labeled multi-Bernoulli filter (GLMB) that models time-variation using labeled RFS [5,6,7]. In all experiments, the tracking involves an unknown and TV number of moving unlabeled objects that enter, leave or stay in the scene at different time steps. In addition, the measurements are unordered and their associations to the objects are unknown. We also add more complexity to the tracking scene, including the presence of clutter, objects moving in close proximity, and varying signal-to-noise ratio (SNR).

Our simulations consider the tracking of multiple objects that are moving in the two-dimensional (2D) plane. For example, the simulated scenario can involve the tracking of an unknown number of cars that move in and out of a busy intersection, where it is possible for a car to make a left or right turn. Unless otherwise stated, the simulations used the following parameters. We assume that there are $N_{k}$ cars moving in the scene at time step *k*. The unknown state parameter vector for the *ℓ*th car is $x_{\ell ,k}$ = $\left[x_{\ell ,k}\;{\dot{x}}_{\ell ,k}\;y_{\ell ,k}\;{\dot{y}}_{\ell ,k}\;\omega_{\ell ,k}\right]$, *ℓ* = $1,\ldots ,N_{k}$, where $\left(x_{\ell ,k},y_{\ell ,k}\right)$ and $\left({\dot{x}}_{\ell ,k},{\dot{y}}_{\ell ,k}\right)$ are the 2-D Cartesian coordinates for the car’s position and velocity, respectively, and $\omega_{\ell ,k}$ is the car’s constant turn rate. The state transition that describes the physics-based model of coordinated turn motion is given by $x_{\ell ,k}$ = $F\;x_{\ell ,k-1}+v_{\ell ,k-1}$, where matrices *F* and $Q_{v}$, the covariance matrix of the zero-mean Gaussian modeling error $v_{\ell ,k-1}$, are:

$$\begin{array}{c} F=\left[\begin{array}{ccccc} 1 & \frac{\sin(\omega_{k-1})}{\omega_{k-1}} & 0 & -\frac{1-\cos(\omega_{k-1})}{\omega_{k-1}} & 0\\ 0 & \cos(\omega_{k-1}) & 0 & -\sin(\omega_{k-1}) & 0\\ 0 & \frac{1-\cos(\omega_{k-1})}{\omega_{k-1}} & 1 & \frac{\sin(\omega_{k-1})}{\omega_{k-1}} & 0\\ 0 & \sin(\omega_{k-1}) & 0 & \cos(\omega_{k-1}) & 0\\ 0 & 0 & 0 & 0 & 1 \end{array}\right],\;\;\;Q_{v}=\left[\begin{array}{ccccc} \frac{\sigma^{2}}{4} & \frac{\sigma^{2}}{2} & 0 & 0 & 0\\ \frac{\sigma^{2}}{2} & \sigma^{2} & 0 & 0 & 0\\ 0 & 0 & \frac{\sigma^{2}}{4} & \frac{\sigma^{2}}{2} & 0\\ 0 & 0 & \frac{\sigma^{2}}{2} & \sigma^{2} & 0\\ 0 & 0 & 0 & 0 & \sigma_{v}^{2} \end{array}\right] \end{array}$$

with σ = 15 m/s^2^ and $\sigma_{v}$ = $\frac{\pi }{180}$ rad/s. The measurements, angle bearing $\phi_{k}\;\;\in \;\;\left(-\frac{\pi }{2},\frac{\pi }{2}\right)$ and range $r_{k}\in \left(0,2\right)$ km, are related to the unknown state parameters according to $z_{k}$ = $\left[\phi_{k}\;r_{k}\right]+w_{k}$ = $h\left(x_{\ell ,k}\right)+w_{k}$, where $h(x_{\ell ,k})$ = $\left[\arctan\;(y_{\ell ,k}/x_{\ell ,k})\;{\left(x_{\ell ,k}^{2}+y_{\ell ,k}^{2}\right)}^{1/2}\right]$. The noise $w_{k}$ is assumed zero-mean Gaussian with covariance matrix $Q_{w}$ = $\mathrm{diag}(25,{\left(\frac{\pi }{180}\right)}^{2})$; the SNR is −3 dB. The maximum number of time steps is 100 and 10,000 Monte Carlo realizations are simulated. For DDP and DPYP, the cluster parameter base distribution $G_{0}$ is generated using a normal-inverse Wishart distribution and the gamma distribution is used as the prior for the concentration parameter α. The probability of object survival is $P_{\ell ,k|k-1}$ = 0.95, ∀ℓ. We use the optimal subpattern assignment (OSPA) metric, with cut-off parameter *c* = 100 and order *p* = 1, to compare the tracking performance [34]. This is a metric associated with tracking multiple objects as it provides both cardinality and state estimation error. Note that the lower the OSPA value the higher the performance.

**Figure 2:**
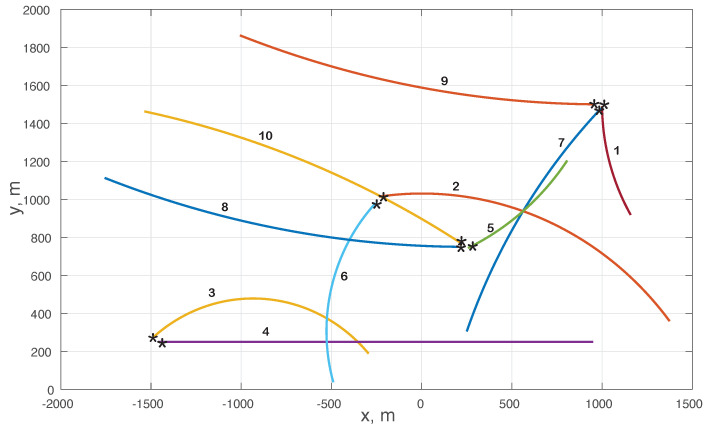
True (x,y)-coordinates for the moving 10 objects in Example 1; the object number and the coordinate at which the object enters the scene (marked by 🟉) are listed in Table 1.

**Experiment** **1.** *DDP-STP for tracking multiple objects in clutter. We consider the tracking of a maximum number of 10 moving objects, similar to the example used for the GLMB (see Section IV.B in [7]). The noisy measurements are assumed to have originated either from the objects or from clutter. In the simulations, we assumed that the number of false alarms follows a Poisson distribution with average ρV = 40, where ρ is the clutter density and *V* is the validation gate volume. Note that the validation gate corresponds to a region in the observation space with measurements validated to have potentially originated from the objects [35]. The clutter model also assumes that the clutter is uniformly distributed in the volume. For each object, Table 1 lists the time steps they enter and leave the scene, together with the (x,y)-coordinates at which they enter the scene. These coordinates are marked by 🟉 in Figure 2; the figure also depicts the true coordinates of the moving objects. For this example, NIW(0.001,0,50,I) was used for $G_{0}$ and Γ(1,0.1) for α; here, I is the identify matrix*.

**Figure 3:**
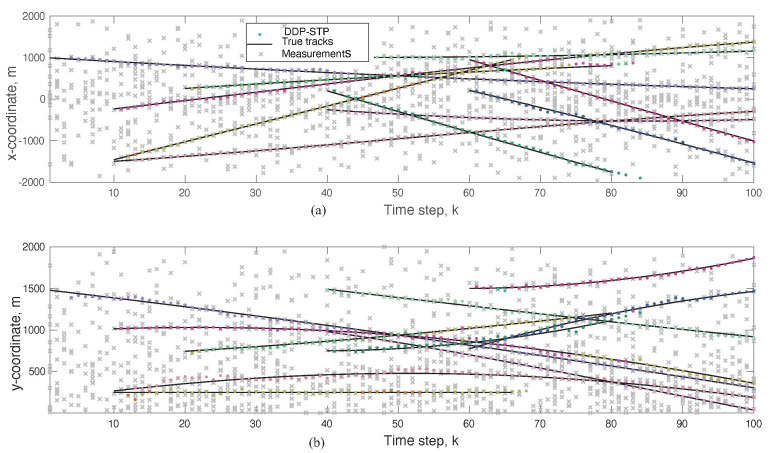
(**a**) $x_{k}$ and (**b**) $y_{k}$ actual and DDP-STP estimated coordinates at time step *k* in Experiment 1. DDP-STP determines that the scattered measurements (marked by *x*) correspond to clutter.

**Figure 4:**
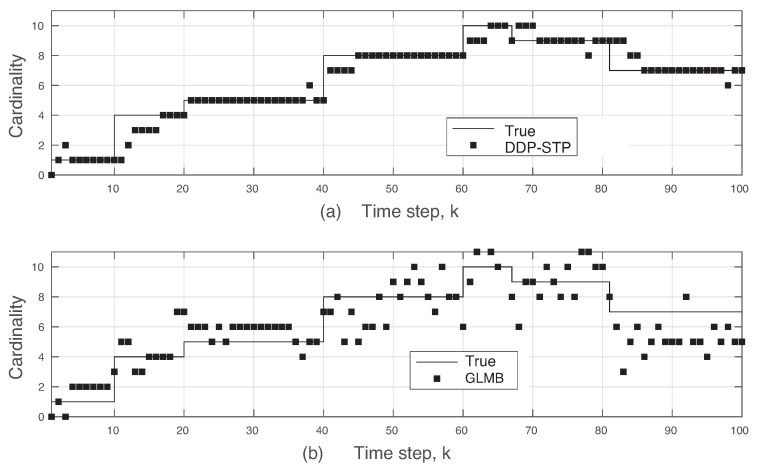
Estimated cardinality using (**a**) DDP-STP and (**b**) GLMB in Experiment 1.

**Figure 5:**
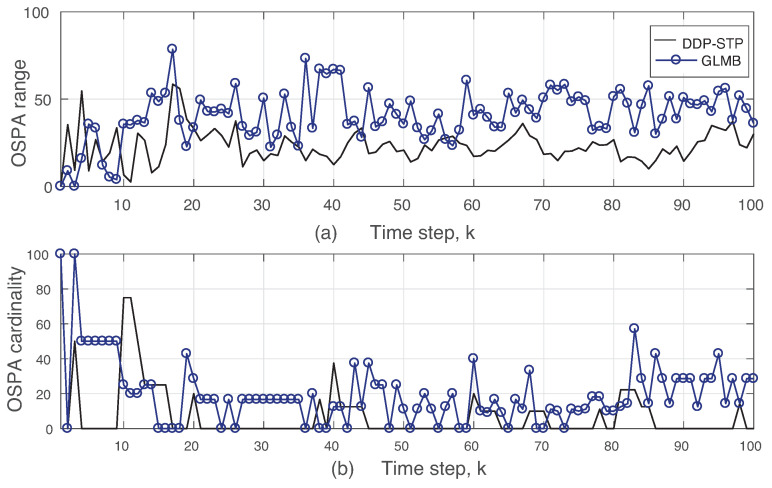
(**a**) OSPA range and (**b**) OSPA cardinality using DDP-STP and GLMB in Expriment 1.

The $x_{k}$ and $y_{k}$ coordinates estimated using the DDP-STP are compared to the true ones in Figure 3a,b, respectively. As the mixing measure used to infer the likelihood distribution in (13) is drawn from the DDP, the DDP-STP identifies the measurements that are not on the tracks as clutter and does not use it to update the object states. Figure 4a,b show the estimated TV object cardinality for the DDP-STP and GLMB, respectively. The OSPA cardinality is also compared in Figure 5b and the OSPA for the estimated range (computed as $\sqrt{x_{k}^{2}+y_{k}^{2}}$) is compared in Figure 5a. As shown, the new DDP-STP method results in higher tracking performance than the GLMB. This is because the GLMB filter is highly sensitive to the presence of clutter as it assumes that clutter statistics are known a priori [36]; this assumption is not needed for the DDP-STP. The GLMB also uses approximations to update the tracks.

**Figure 6:**
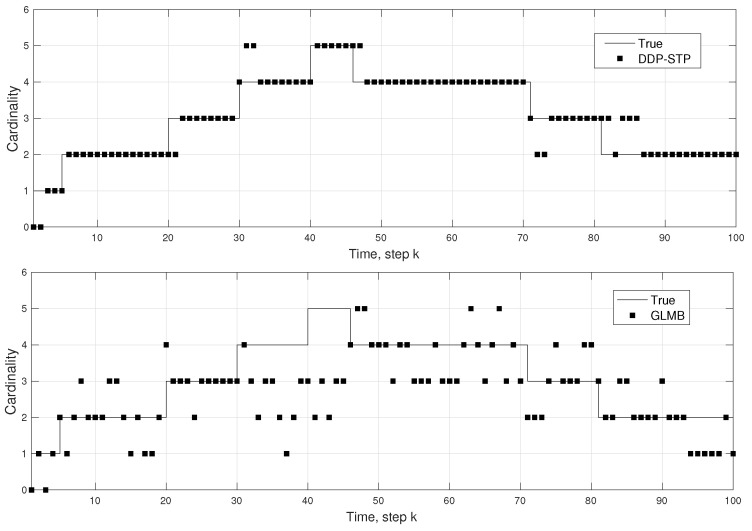
True and estimated cardinality using (**a**) DDP-STP and (**b**) GLMB in Experiment 2.

**Figure 7:**
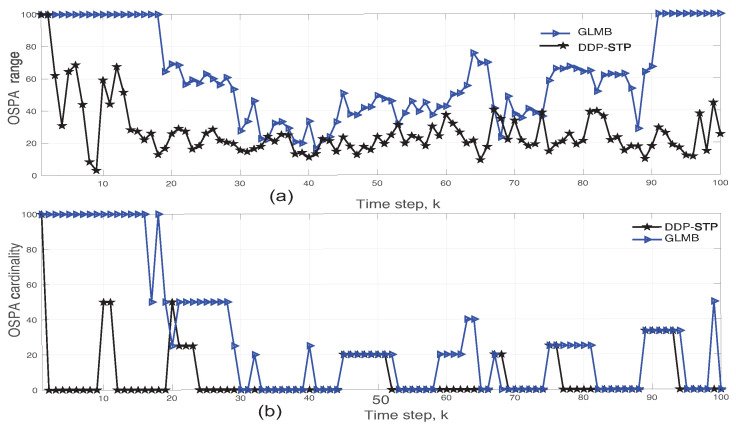
OSPA (**a**) range and (**b**) cardinality using DDP-STP and GLMB in Experiment 2.

**Experiment** **2.** *DDP-STP for tracking multiple objects in close proximity. We consider a more complex scenario, where objects are moving in close proximity to each other. A maximum of 5 objects enter the scene at times steps k = 0, k = 5, k = 20, k = 30, and k = 40, respectively; they leave the scene at time steps k = 70, k = 100, k = 100, k = 45, and k = 80. All 5 objects follow the same path but at different times. For this experiment, NIW(0.001,0,100,I) was used for $G_{0}$ and Γ(1,0.3) for the concentration parameter prior. The comparison between the DDP-STP and GLMB for the estimated cardinality and OSPA metrics are shown in Figure 6 and Figure 7, respectively. As demonstrated, the DDP-STP performs much higher than the GLMB for closely-spaced targets*.

**Figure 8:**
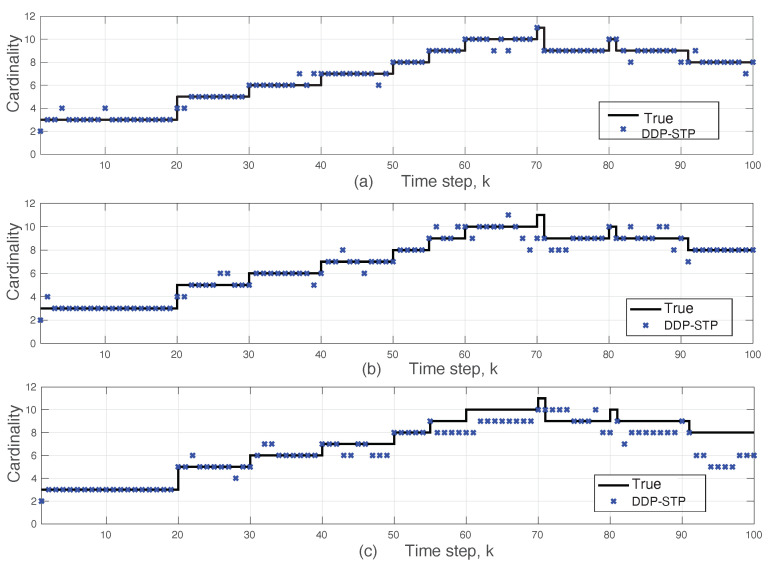
Comparison of true and estimated object cardinality using DDP-STP for (**a**) −3 dB, (**b**) −5 dB, and (**c**) −10 dB SNR in Experiment 3.

**Figure 9:**
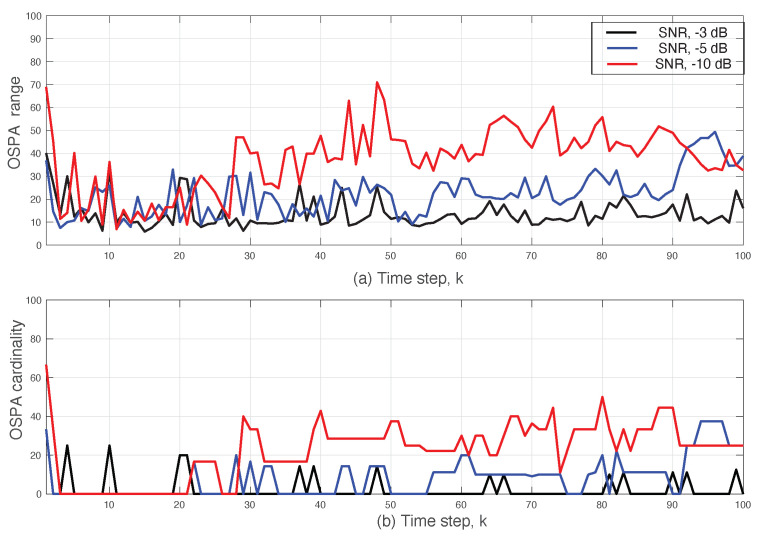
DDP-STP OSPA (**a**) range and (**b**) cardinality for −3, −5 and −10 dB SNR in Experiment 3.

**Experiment** **3.** *DDP-STP for tracking multiple objects under varying SNR. We demonstrate the effect of varying the SNR when tracking multiple targets using DDP-STP. In this experiment, we assume that 11 objects enter and leave the scene at different times, as shown by the true object cardinality in Figure 8. We use $\omega_{k}$ = 0 and assume that only range measurements are available. The tracking was simulated for −3 dB, −5 dB and −10 dB SNR using NIW(0,0,100,I) for $G_{0}$ and Γ(1,0.2) for α. The estimated cardinality is compared to the true one for decreasing SNR in Figure 8a–c. As expected, the performance of the DDP-STP decreases as the SNR decreases. Figure 9a,b compare the OSPA range and OSPA cardinality performance as the SNR decreases*.

**Figure 10:**
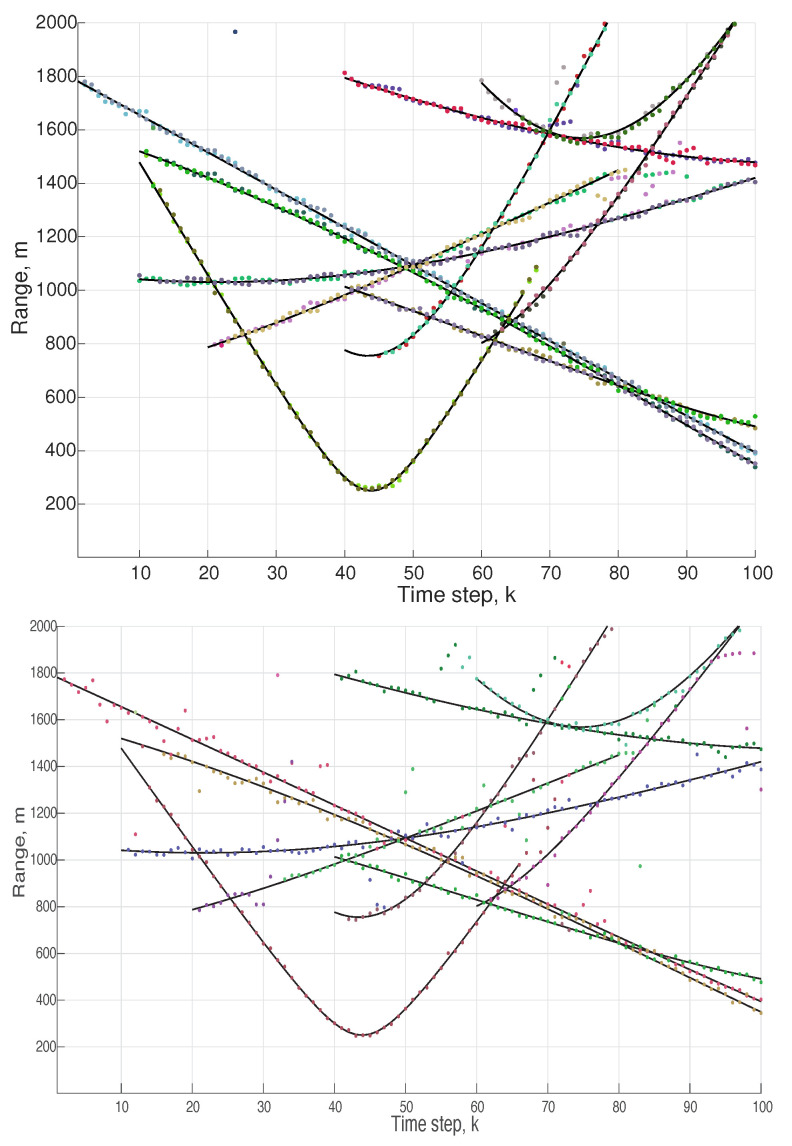
Range estimation using DPY-STP (**top**) and DDP-STP (**bottom**) in Experiment 4.

**Figure 11:**
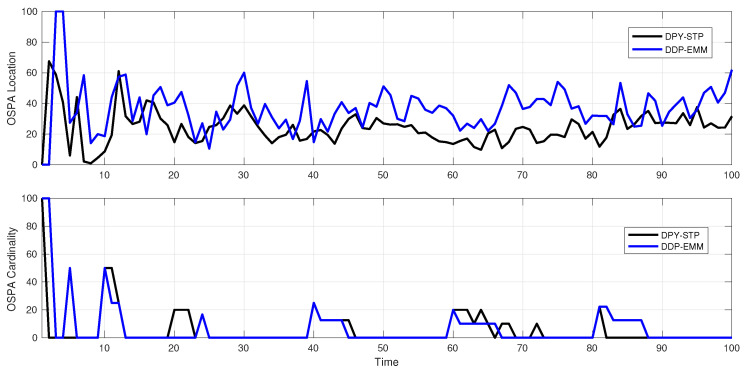
OSPA comparison between DPY-STP (black) and DDP-STP (blue).

**Experiment** **4.** *DPY-STP and DDP-STP for tracking multiple objects. As discussed in Section 4, the DPYP is a better match than the DDP when tracking objects with high variability in the scene. This is demonstrated by comparing the new DPY-STP and DDP-STP methods in tracking a maximum number of 10 targets. The simulations used NIW(0,0,100,I) for $G_{0}$ and Γ(1,0.1) for α for both methods. Using empirical Bayes, the DPYP discount parameter value was approximated to d = 0.37. The increased performance of the DPY-STP is demonstrated by comparing the true and estimated range obtained by the DPY-STP and DDP-STP in Figure 10. The increased performance is attributed to the increased flexibility of the DPYP in dynamically selecting clusters with a large time-varying number of objects moving in the tracking scene. We also demonstrate this using the OSPA metric with cut-off c = 100 and order p = 1. We observe that DPY-STP has a better performance compared to DDP-STP as depicted in Figure 11*.

## 6. Conclusions

We proposed new methods for tracking multiple objects under various unknown conditions. In particular, the number of moving objects is unknown and varies with time, as objects can enter, leave or remain in the tracking scene at any time. Furthermore, the measurements are unordered and the measurement-to-object associations are unknown. The methods integrate Markov chain Monte Carlo methods with dependent Bayesian nonparametric models to account for dynamic dependencies in the tracking formulation. Specifically, the proposed DDP-STP tracking algorithm exploits the dynamic clustering property of the dependent Dirichlet process (DDP) to learn unlabeled information in the state transition formulation. Dirichlet process mixtures are used to associate measurements to objects, drawing the mixing mixtures from DDP to learn the likelihood distribution. The Bayesian posterior used to obtain the object state estimates is efficiently implemented using a Gibbs sampler inference scheme. The second proposed tracking method, DYP-STP uses the dependent Pitman–Yor (DPY) process. The DPY-STP is advantageous over the DDP-STP when higher variability in the dynamic clustering is required to handle the higher variability in the tracking formulation.

We used simulations to compare the DDP-STP with the generalized labeled multi-Bernoulli (GLMB). We demonstrated that the dynamic clustering offered by the DDP is more flexible to object labeling in addition to identifying measurement-to-object associations using DP mixtures. Allowing for a dynamically varying number of clusters, the new methods perform well even in the presence of clutter measurements without knowledge of clutter statistics needed by the GLMB. Furthermore, unlike the GLMB, the new methods do not require any approximations in solving rank assignments. This allows for more efficient implementation in the new methods as well as their applicability in tracking a large number of objects in multimodal sensing systems.

## Figures and Tables

**Table 1:** Enumerated objects in Experiment 1 with time steps at which *ℓ*th object enters and leaves the scene and (x,y)-coordinate at which object enter the scene.

Object Number *ℓ*	Time Step *k* Object Enters	Time Step *k* Object Leaves	(*x,y*) m That Object Enters
1	0	100	(1000, 1488)
2	10	100	(−245, 1011)
3	10	100	(−1500, 260)
4	10	66	(−1450, 250)
5	20	80	(245, 740)
6	40	100	(−256, 980)
7	40	100	(950, 1470)
8	40	80	(230, 740)
9	60	100	(930, 1500)
10	60	100	(220, 750)

## Data Availability

The data generated in this paper are as described in the paper. For more information regarding this paper, one can check https://bmoraffa.github.io (accessed on 1 December 2021).
